# Supplementary material for: Study protocol for the ‘HelpMeDoIt!’ randomised controlled feasibility trial: an app, web and social support-based weight loss intervention for adults with obesity
Source: BMJ Open. 2017 Oct 25;7(10):e017159. doi: 10.1136/bmjopen-2017-017159 (PMC5665248; doi:10.1136/bmjopen-2017-017159)
Supplement: Supplementary data [file bmjopen-2017-017159supp002.pdf]

Appendix 2. Informed Consent Form

HelpMeDoIt! Stage 2 Participant Consent Form

Part 1: TAKING PART AND USE OF MY INFORMATION FOR THIS PROJECT

Please initial box

1. I confirm that I have read and understood the Stage 2 Participant Information Sheet dated 28/01/16 (Version 1.2) for the above study. I have had the opportunity to consider the information, ask questions and have had these answered to my satisfaction. ☐

2. I understand that my participation is voluntary and that I am free to withdraw at any time, without giving any reason, and without my medical care or legal rights being affected. ☐

3. I understand that information about me needed for the study (including personally identifiable information) may be collected from me and looked at by the research team from The University of Glasgow during the study. It may also be looked at by regulatory authorities supervising the study, and the NHS Board, where it is relevant to my taking part in this research. I give permission for these individuals to have access to this. ☐

4. I give permission for my GP to be informed of my participation in this study. ☐

5. I understand that my words may be quoted in publications, reports, web pages and other research outputs. This will be anonymous and I won't be identified from any of the outputs. ☐

6. I agree to take part in the above study. ☐

Name of Participant	Date	Signature of Participant
Name of Researcher	Date	Signature of Researcher

Part 2: USE OF MY INFORMATION BEYOND THIS PROJECT

7. I agree to be contacted by the Social and Public Health Sciences Unit (University of Glasgow) about participating on future similar studies. ☐

8. I agree for the data I provide to be anonymously archived in the UK Data Archive. ☐

9. I understand that other genuine researchers will have access to this data only if they agree to preserve the confidentiality of the information as requested in this form. ☐

10. I understand that other genuine researchers may use my words in publications and other outputs only if they agree to preserve the confidentiality of the information as requested in this form. ☐

11. I agree to my information being shared anonymously with other researchers if they have scientific and ethical approval for the questions that they would like to answer with the information, and have the right training.. ☐

____________________	________________	____________________
Name of Participant	Date	Signature of Participant
__________________	________________	____________________
Name of Researcher	Date	Signature of Researcher

When completed please place the top copy in the site file and give one copy to the participant.
