# Supplementary figures and images for: Study protocol for the ‘HelpMeDoIt!’ randomised controlled feasibility trial: an app, web and social support-based weight loss intervention for adults with obesity
Source: BMJ Open. 2017 Oct 25;7(10):e017159. doi: 10.1136/bmjopen-2017-017159 (PMC5665248; doi:10.1136/bmjopen-2017-017159)

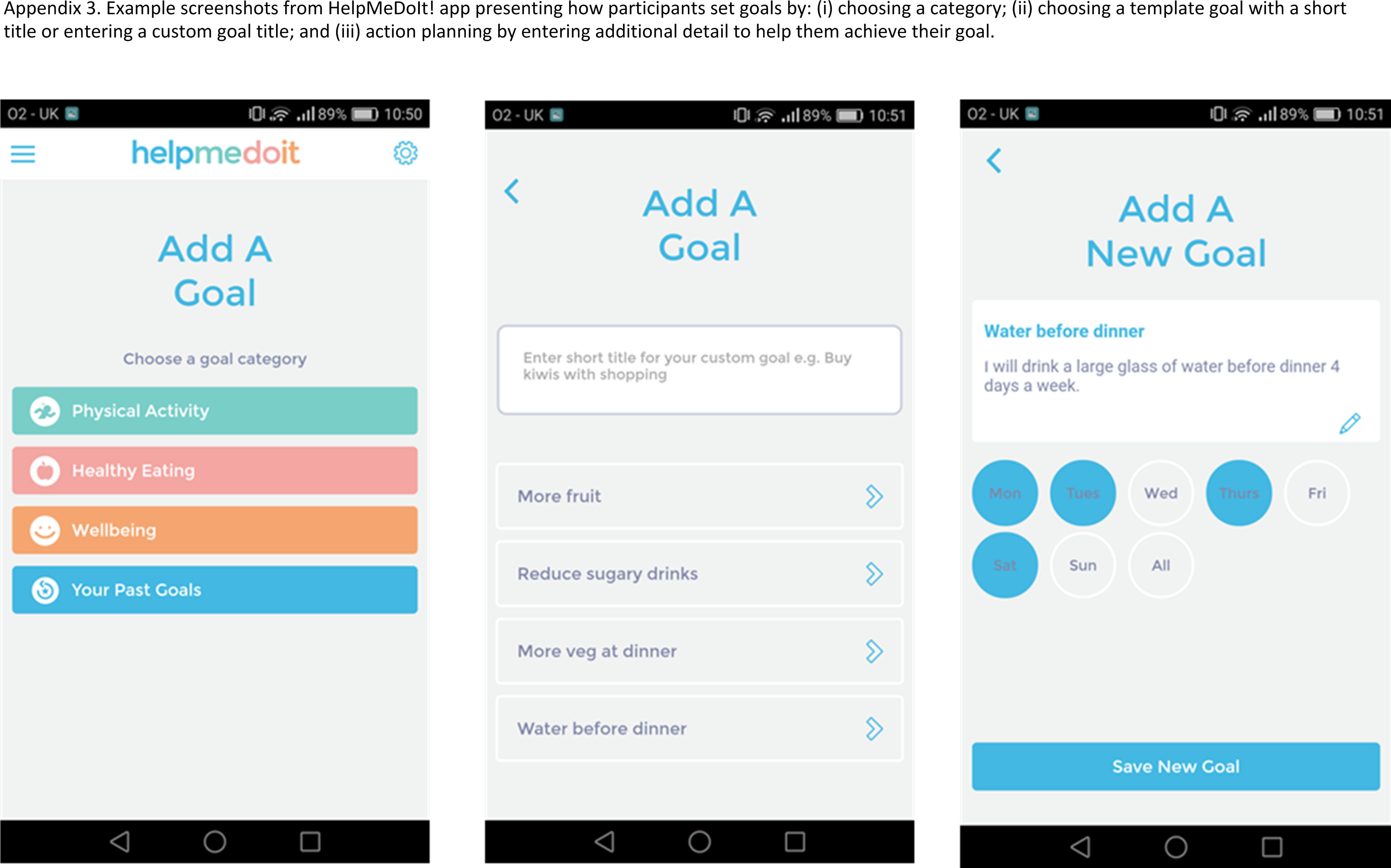

Supplement: Supplementary data [file bmjopen-2017-017159supp003.jpg]

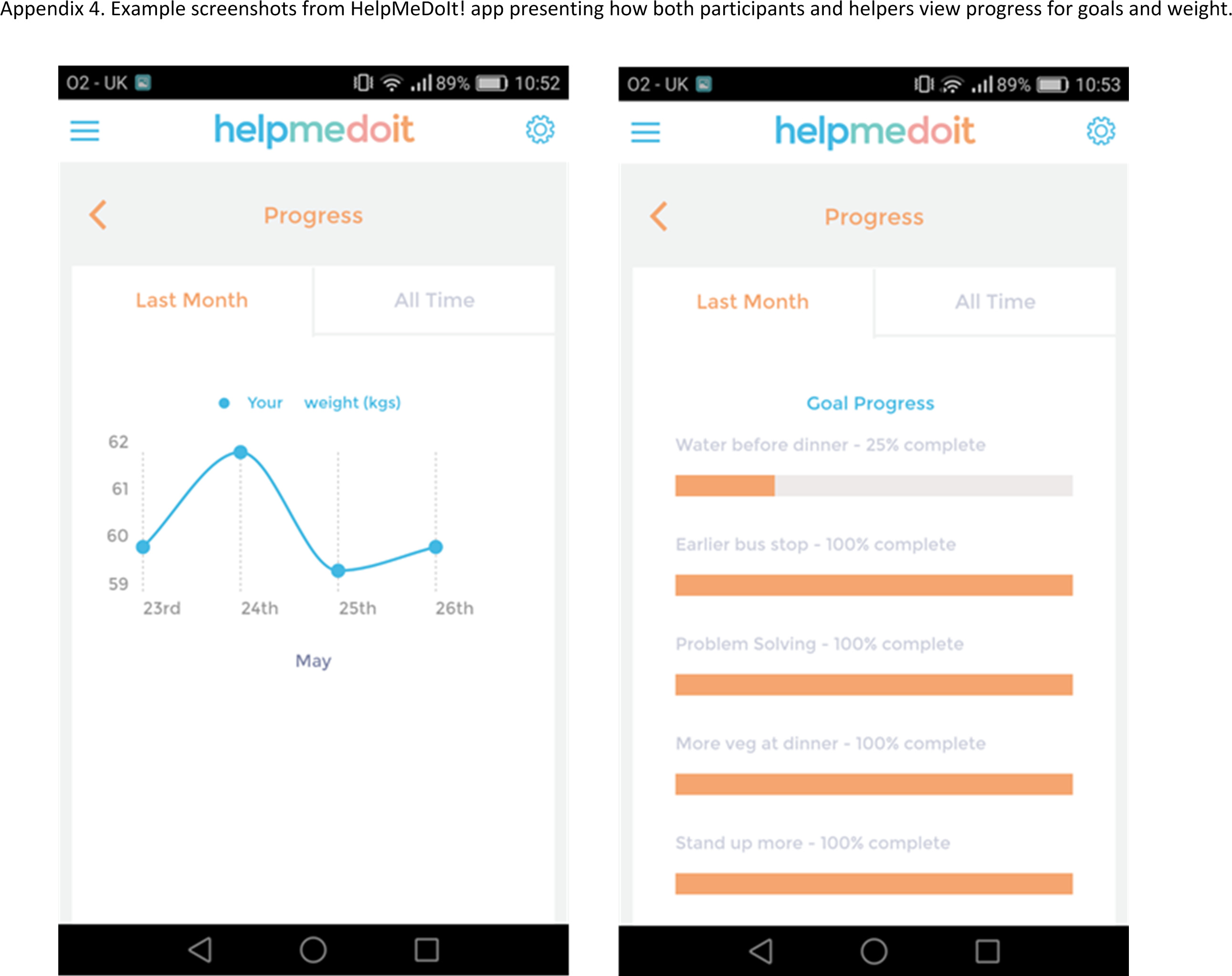

Supplement: Supplementary data [file bmjopen-2017-017159supp004.jpg]

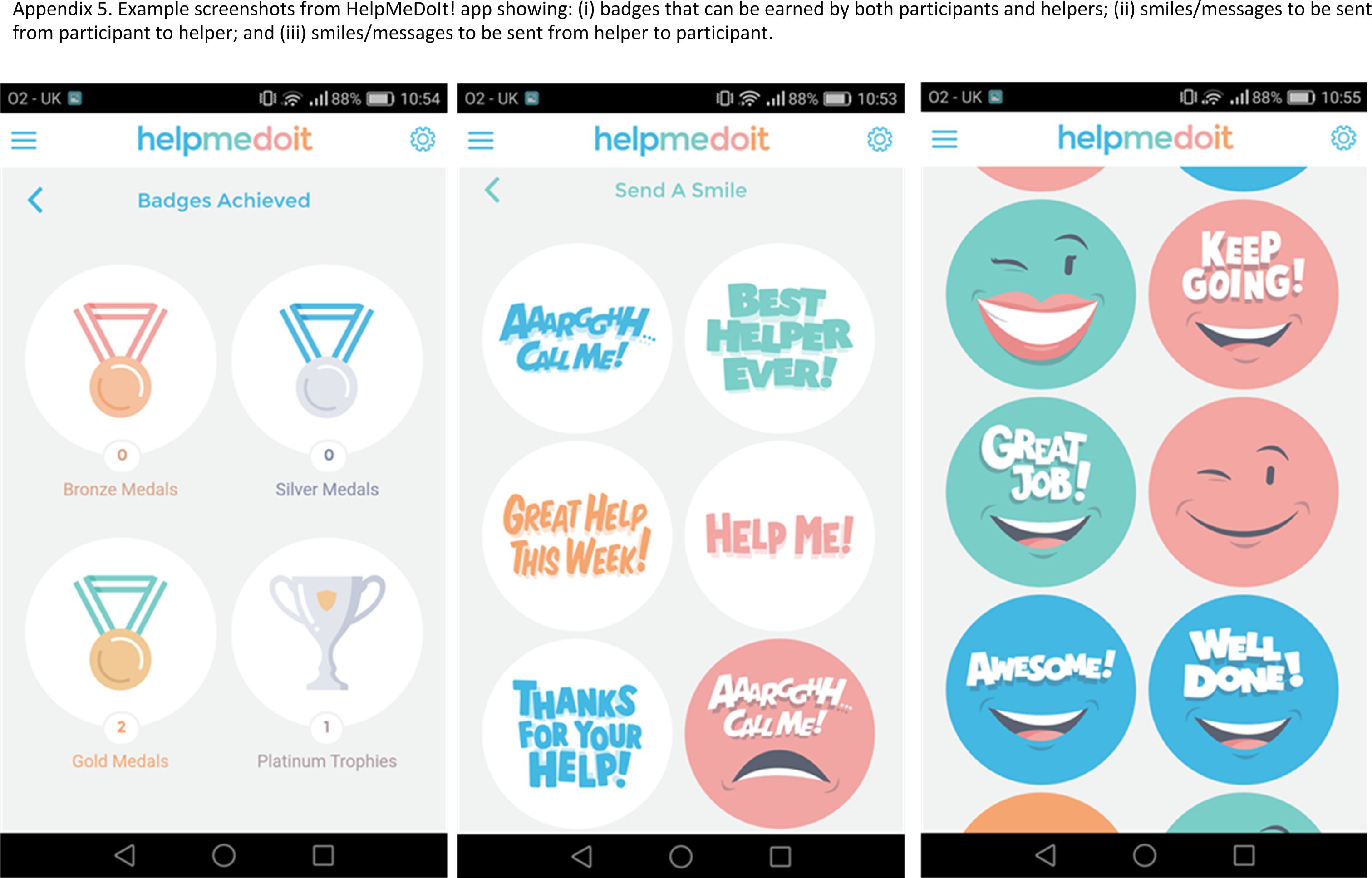

Supplement: Supplementary data [file bmjopen-2017-017159supp005.jpg]

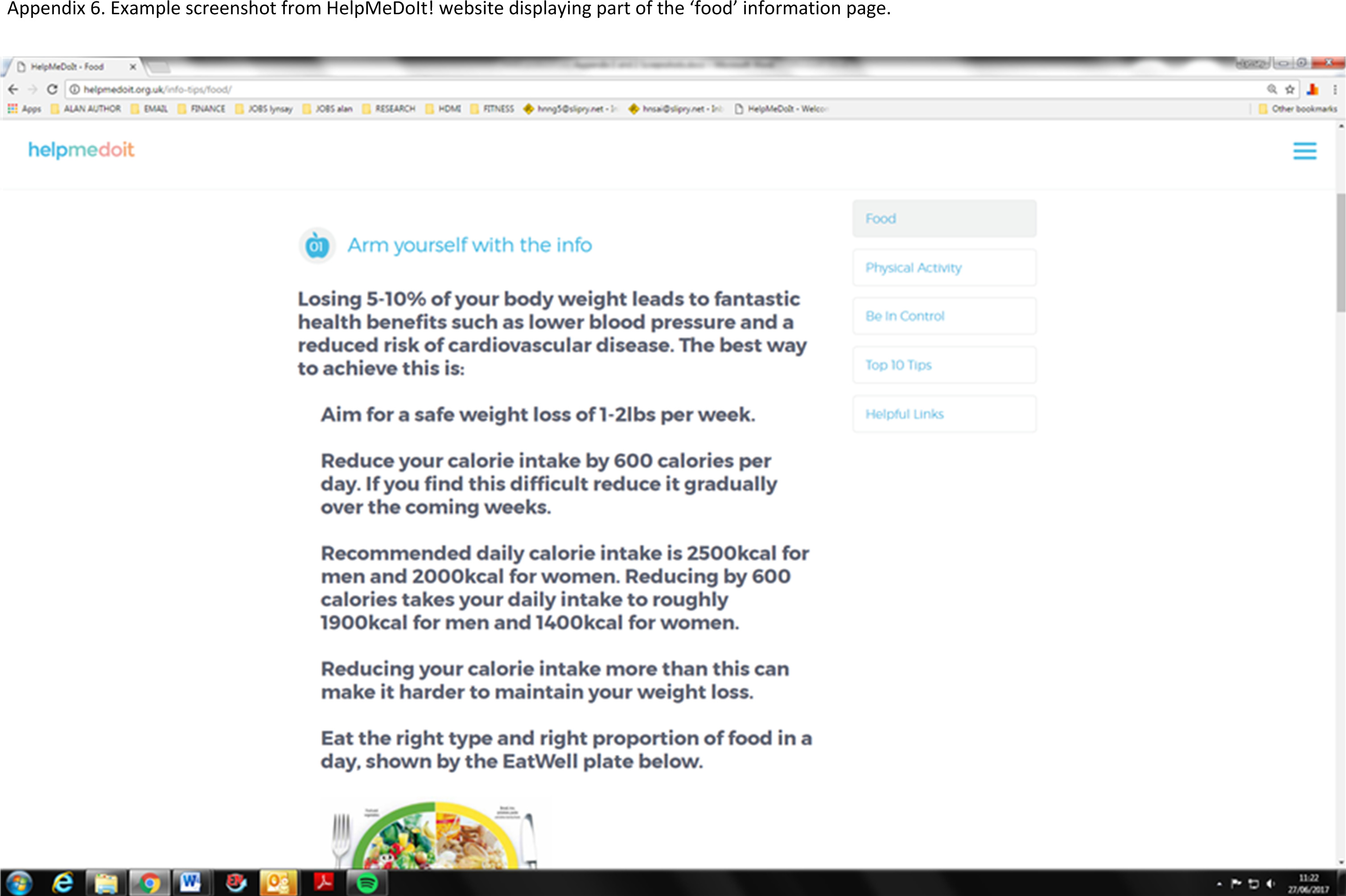

Supplement: Supplementary data [file bmjopen-2017-017159supp006.jpg]

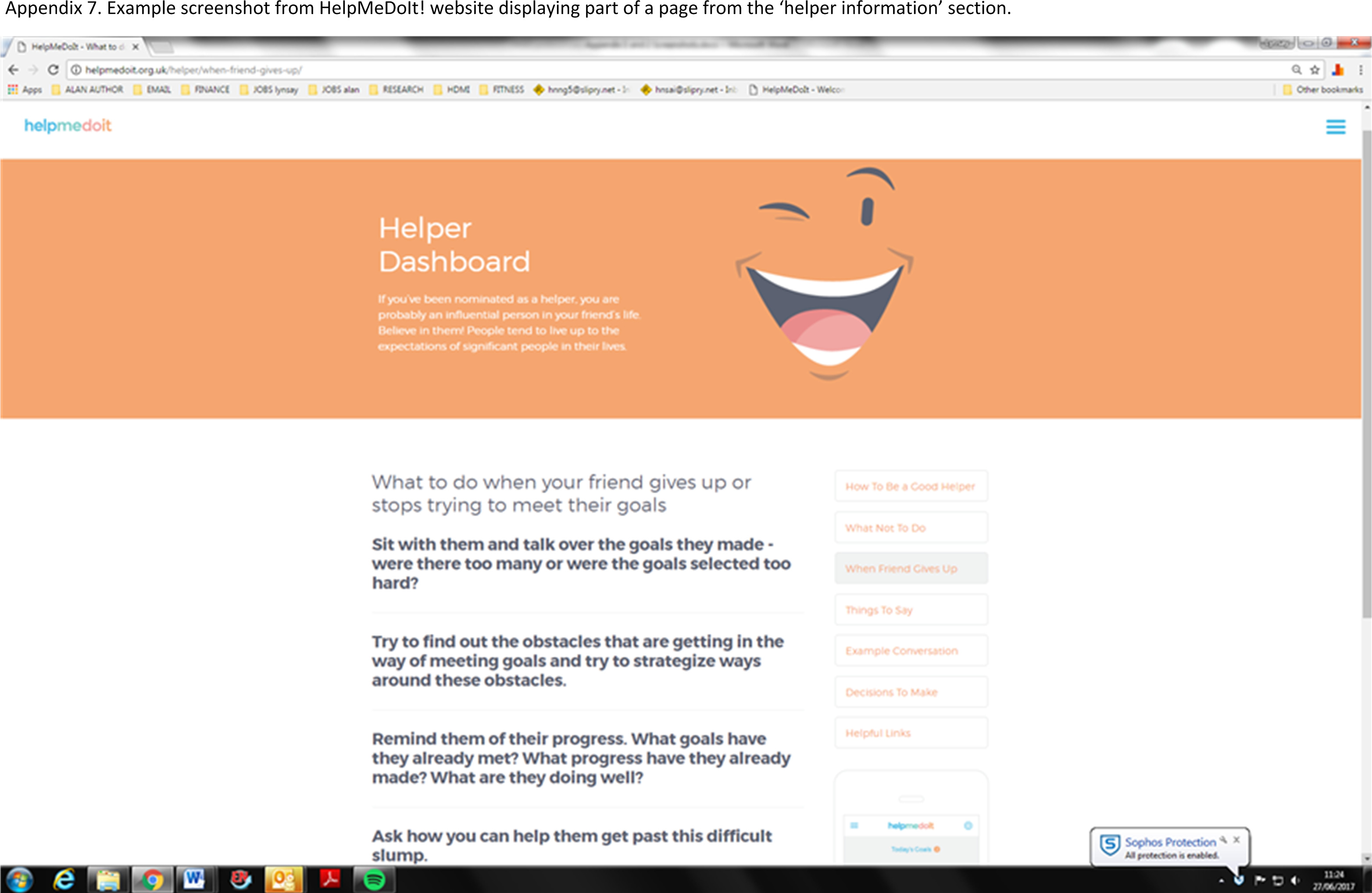

Supplement: Supplementary data [file bmjopen-2017-017159supp007.jpg]
